# Molecular Characterization of Acyl-CoA Oxidase (ACX) Family Genes in Maize Reveals Their Role in Disease Resistance

**DOI:** 10.3390/genes16050486

**Published:** 2025-04-25

**Authors:** Ruobing He, Wenxiao Ma, Fan Zhou, Hongzhe Cao, Kang Zhang, Jingao Dong, Jihong Xing

**Affiliations:** 1 State Key Laboratory of North China Crop Improvement and Regulation, Hebei Agricultural University, Baoding 071000, China; heruobing163@163.com (R.H.);; 2 Hebei Key Laboratory of Plant Physiology and Molecular Pathology, Hebei Agricultural University, Baoding 071000, China

**Keywords:** maize, ACX family genes, expression pattern, biotic stress, abiotic stress

## Abstract

Background: Acyl-CoA oxidase (ACX), a ubiquitous eukaryotic enzyme, catalyzes the initial steps of fatty acid β oxidation and plays an important role in the biosynthesis of jasmonic acid (JA). At present, no studies have been reported on ACX family members of maize and their function in disease resistance. Objectives: This study aims to lay a foundation for clarifying the functions of ACX family genes in maize growth, development, and stress response by conducting a genome-wide identification of ACX family genes in maize, analyzing the expression characteristics of these genes in maize growth and development, hormone treatment and response to biotic and abiotic stresses, and exploring the functions of key genes in the maize disease resistance process through the use of mutants. Methods: ProtParam, TBtools, MEME, MEGA, and IBS tools were used to identify maize ACX family genes and analyze the physicochemical properties of their proteins, chromosome location, phylogenetic relationships among family members, conserved domains, conserved motifs, and cis-acting elements. Meanwhile, the expression patterns of maize ACX family genes in different tissues and their expression patterns under abiotic and biotic stresses were studied by using the data from the maize GDB database and qRT-PCR technology. Moreover, the mutants of *ZmACX1*, *ZmACX3*, *ZmACX4*, and *ZmACX5* genes were obtained, and the disease resistance of the mutants was detected to further determine the functions of ACX genes in the maize disease resistance process. This study identified maize ACX family genes using bioinformatics methods. Results: We discovered that six ACX genes in the maize genome are distributed across four different chromosomes. Cluster analysis further classified these genes into three subfamilies. All maize ACX genes possess a conserved ACOX domain, and their promoter regions are enriched with cis-acting elements associated with heat stress and the plant hormone response. Under various tissue, biotic, and abiotic stress conditions, as well as treatments with methyl jasmonate (MeJA) and salicylic acid (SA), the expression levels of maize ACX family genes exhibited significant differences. Notably, the expression levels of *ZmACX1*, *ZmACX3*, *ZmACX4*, and *ZmACX5* were significantly up-regulated following stress and pathogen infection, suggesting their involvement in maize growth, development, and disease resistance. To elucidate the function of these genes in maize disease resistance, the resistance of *ZmACX1*, *ZmACX3*, *ZmACX4*, and *ZmACX5* mutants to *Cochliobolus heterostrophus*, *Curvularia lunata*, and *Fusarium graminearum* were further examined. The results revealed that compared to the wild-type B73, the lesion area of the mutants was significantly increased after inoculation with pathogens. This directly demonstrated the crucial role of these genes in maize resistance to *C. heterostrophus*, *C. lunata*, and *F. graminearum*. Conclusions: In summary, this study systematically identified maize ACX family genes, and thoroughly investigated their expression patterns and functions in maize disease resistance. Our findings provide valuable insights into the comprehensive understanding of the function and mechanism of maize ACX family genes.

## 1. Introduction

Maize (*Zea mays*), the world’s foremost food crop, has significantly contributed to global food security [1], improving farmers’ income and facilitating animal husbandry development. As ecological challenges escalate, the demand for high-yield and high-quality maize varieties is on the rise, prompting deeper exploration into maize’s mechanisms for coping with environmental stresses. Jasmonic acid (JA) and SA are vital plant hormones that play pivotal roles in plant growth, development, and defense response [2,3]. JA, as a resistance hormone, is crucial for plant resistance to biotic and abiotic stresses. It stimulates the expression of defense genes and triggers defensive mechanisms in plants to counteract mechanical injury or insect herbivory [4]. In unstressed plants, the JAV1-JAZ-WEKY51 complex inhibits the expression of JA biosynthesis genes, maintaining low JA levels to support normal plant growth. However, upon insect attack, the calmodulin in JAV1 becomes phosphorylated, leading to the degradation of the JJW complex and the activation of JA biosynthesis, resulting in a surge in JA content that aids in plant defense [5]. SA, an essential endogenous signaling molecule in plant stress responses, activates plant hypersensitive response, thereby inducing resistance to exogenous stresses [6]. When a plant part is infected by pathogens, SA promotes antibody production in other parts of the plant and facilitates inter-plant communication through volatile methyl salicylate (MeSA) to collectively resist pathogen infection. Furthermore, SA regulates physiological processes such as plant growth, development, maturation, and senescence, and is involved in anti-salt, anti-drought, anti-low temperature, anti-ultraviolet, and anti-heavy metal stress reactions [7,8,9].

Acyl-CoA oxidase (ACX) is a peroxisomal flavoprotein involved in signal transduction pathways, including receptor activation, fatty acid metabolism, and polyunsaturated fatty acid synthesis [10,11]. As a key enzyme in the β-oxidative metabolic pathway, ACX is ubiquitous in animals, plants, and microorganisms. β-oxidation of fatty acids is the primary metabolic pathway for rapidly degrading reserve lipids and providing carbon sources and energy during growth and development. In plants, peroxisomal β-oxidation plays a predominant role, decomposing various fatty acid forms from short to long chains, particularly during germination [12]. ACX catalyzes the first rate-limiting reaction in β-oxidation, the oxidation of fatty acid acyl-CoA to 2-trans-olefin-CoA. Enhanced fatty acid β-oxidation is crucial for early seedling growth post-germination [13].

Studies have shown that in oilseeds, peroxisomal β-oxidation influences triacylglycerol transformation, membrane lipid turnover, aging, and starvation processes [14]. In soybean (*Glycine max*), other GmACX1 and GmACX2 accumulated in seedling tissues, with stronger expression in the growing seedling axis and hypocotyl, but weaker in the cotyledon [15]. In Arabidopsis (*Arabidopsis thaliana*), *acx1* and *acx2* double-mutants exhibit growth defects early in seedling development, whereas single *acx* mutants do not show such deficiencies [16]. These findings indicate that ACX isoenzymes may synergistically affect plant growth and development.

Plants possess multiple ACX isoenzymes with varying sizes and subunit composition, classified into three categories based on the carbon chain length they recognize in catalytic reactions: long-chain ACX (LACX), medium-chain ACX (MACX), and short-chain ACX (SACX) [17,18]. ACX1 prefers long-chain saturated and unsaturated CoA, ACX2 exhibits stronger catalytic activity toward long-chain unsaturated CoA than saturated CoA, ACX3 recognizes medium-chain fatty acids, and ACX4 recognizes short-chain fatty acids, while the functions of ACX5 and ACX6 remain unclear [19,20].

ACX participates in diverse plant physiological activities, playing a critical role not only in fatty acid catabolism, but also in the three β-oxidation cycles of the JA synthesis pathway. Thus, ACX is involved in regulating plant growth, development, and stress response. In Arabidopsis, *AtACX1* and *AtACX5* are implicated in JA synthesis [21,22]. An ACX isoenzyme that induces JA synthesis was identified in tomato (*Lycopersicon esculentum*) mutants [23]. In rice (*Oryza sativa*), *OsACX1* is strongly induced by JA, enhancing resistance to rice blast fungus and participating in defense responses to injury and disease [24]. Mutation of ACX1 in Chinese cabbage (*Brassica campestris*) reduces JA levels, affecting the transcription of JA-responsive genes and ultimately leading to petal degradation [25]. In tea plant (*Camellia sinensis*), *CsACX1* and *CsACX3* are associated with JA biosynthesis, and *CsACX3* is induced by *Colletotrichum gloeosporioides*, increasing ACX enzyme activity [26,27]. In Arabidopsis, the activity of AtACX2 and AtACX3 enhances plant defense against necrotic pathogen infections [28].

Although the identification and function of the ACX gene family have been studied in Arabidopsis, rice, tomato, and tea plant, there are no reports on this gene family in maize. In this study, we utilized bioinformatics to identify ACX gene family members in the maize inbred line B73, comprehensively analyzing their physicochemical properties, phylogenetic relationships, conserved domains, motifs, and cis-acting elements. This provides a theoretical foundation for related studies on the maize ACX gene family. Additionally, we systematically analyzed tissue-specific expression and expression patterns under biotic and abiotic stresses. Using qRT-PCR, we further examined the expression of maize ACX family members in response to MeJA and SA treatments. With the aid of the NCBI SRA database of RNA-Seq data and qRT-PCR, we conducted an in-depth analysis of maize ACX family member expression patterns in different tissues, and their responses to exogenous hormone signals and pathogen infections. Furthermore, we assessed the disease resistance of maize ACX gene mutants that significantly respond to stress and pathogens, laying the groundwork for further investigation into the disease resistance function and molecular mechanism of the maize ACX gene family.

## 2. Materials and Methods

### 2.1. Test Materials

Maize inbred line B73 seeds, *C. heterostrophus*, *C. lunata*, and *F. graminearum* strain PH-1, were provided by the Key Laboratory of Plant Physiology and Molecular Pathology of Hebei Province. Prior to the experiment, maize seeds were surface-disinfected and soaked in sterile water for 24 h. The seeds were then grown in an artificial climate chamber at 25 °C to ensure optimal growth conditions. Mutant seeds for *ZmACX1*, *ZmACX3*, *ZmACX4*, and *ZmACX5* were obtained from the Maize Mutant Library (China Mu) using Mutator transposon mutagenesis (B73).

### 2.2. Identification of Maize ACX Family Genes

Maize whole-genome data were downloaded from the Maize GDB [https://www.maizegdb.org/ (accessed on 8 July 2024)] Using the Pfam database (search number PF01756), the ACOX structure domain hidden Markov model was downloaded. The Hmmsearch function in HMMER 3.0 was used to identify all members of the ACX gene family in maize. SMART software was employed to further confirm the conservative domain structure of all identified ACX family members in maize.

### 2.3. Physicochemical Properties and Chromosomal Localization of Maize ACX Proteins

The molecular weight, isoelectric point, and physicochemical properties of maize ACX family members were analyzed using ExPASy [https://www.expasy.org/ (accessed on 8 July 2024)] Chromosome localization analysis and gene density calculation were performed using MG2C [http://mg2c.iask.in/mg2c_v2.1/ (accessed on 8 July 2024)]. The results were visualized using TBtools2.136 software.

### 2.4. Phylogenetic Analysis of Maize ACX Family Genes

Protein sequences of AtACX family members were downloaded from the Thale cress TAIR database [https://www.arabidopsis.org/ (accessed on 14 August 2024)]. Similarly, the protein sequences of the OsACX family members were obtained from the rice RAP-DB database [https://rapdb.dna.affrc.go.jp/ (accessed on 14 August 2024)]. ClustalX2.0 software was used to compare and analyze the amino acid sequences of maize ACX with those of Thale cress and rice. A phylogenetic tree was constructed using MEGA11.0 software with the neighbor-joining (NJ) method, and the bootstrap value was set to 1000.

### 2.5. Prediction Analysis of Maize ACX Conserved Domains and Motifs

The domains of maize ACX members were analyzed using SMART [http://smart.embl-heidelberg.de/ (accessed on 20 August 2024)], and the results were visually analyzed with TBtools software. The conserved motifs of maize ACX family members were analyzed using MEME [https://meme-suite.org/meme/ (accessed on 20 August 2024)]. The number of motifs was set to 10, with other parameters remaining at default settings. The conserved motifs were visualized using TBtools software.

### 2.6. Analysis of Promoter Cis-Acting Elements of Maize ACX Family Genes

The promoter regions of maize ACX members were analyzed using the Plant CARE online tool [http://bioinformatics.psb.ugent.be/webtools/plantcare/html/ (accessed on 25 August 2024)]. The promoter composition results for each member were visually analyzed using IBS software.

### 2.7. Analysis of Tissue Expression Specificity of Maize ACX Family Genes

RNA-Seq data from various maize tissues (root, stem, leaf, tassel, anther, pollen, cob, style, ovule, embryo, endosperm, and seed) were downloaded from the NCBI SRA database [https://www.ncbi.nlm.nih.gov/sra (accessed on 27 August 2024)]. The RNA-Seq sequences were aligned to the maize reference genome using Hisat2 2.2.1 software. Cufflinks 2.2.1 software was used to calculate the gene expression values in FPKM (fragments per kilobase of exon per million fragments mapped reads), considering gene length and read number. The expression heat map of maize ACX family genes was generated using the Heat Map function in TBtools software, visually demonstrating the differences in ACX gene expression across various maize tissues.

### 2.8. Analysis of Expression Patterns of Maize ACX Family Genes Under Biotic and Abiotic Stress

RNA-Seq data from various maize tissues at different developmental stages were obtained from the NCBI SRA database. Abiotic stress conditions included high-temperature treatment (50 °C for 4 h), low-temperature treatment (5 °C for 16 h), salt stress (irrigation with 300 mmol/L NaCl for 20 h), ultraviolet irradiation (2 h), and drought treatment (seedlings placed on and covered with dry filter paper for 4 h). Biotic stresses included the fungal infection of maize leaves 20–22 days after pollination by *Fusarium verticillioides* and *Aspergillus flavus*, and the inoculation of maize stems with *F. graminearum* at the 10-leaf stage. HiSAT2 software was employed to align the RNA-Seq sequences to the maize reference genome, and Cufflinks 2.2.1 was utilized to calculate gene expression values in FPKM.

### 2.9. Analysis of Expression Patterns of Maize ACX Family Genes Under Exogenous Hormone Treatment

Maize B73 plants at the 3-leaf stage were treated with MeJA (0.1 mmol/L) and SA (0.1 mmol/L). Samples were collected at 0, 3, 6, 12, and 24 h after treatment. Total RNA was extracted from the sample using a plant RNA extraction kit (OMEGA, Norcross, GA, USA) and reverse-transcribed using the TaKaRa Prime Script^TM^ RT kit. The maize tubulin gene served as the reference, and specific primers of maize ACX family genes (Table S1) were used for qRT-PCR analysis. The experiment was repeated three times for each sample, and the 2^−ΔΔCt^ method was used to analyze the expression levels of target genes under different hormone treatment conditions, and the results were visualized using GraphPad Prism9.

### 2.10. Disease Resistance Analysis of Maize ACX Family Genes

Four maize ACX gene mutants (*ZmACX1*, *ZmACX3*, *ZmACX4*, and *ZmACX5*) were selected based on their response to biotic and abiotic stress. The disease resistance of these mutants was analyzed using *C. heterostrophus*, *C. lunata*, and *F. graminearum*. The fungal strains were cultured on a PDA medium at 28 °C for 7–10 days until they covered the entire dish. Wild-type B73 and mutant maize plants were grown to the 7-leaf stage, and the 4th and 5th leaves were selected for inoculation. A sterile needle was used to scratch the maize leaves lightly for 2–4 mm. A 200 µL aliquot of diluted T20 was pipetted onto the incision, and the fungal cultures were placed upside down on the leaf wounds. The plants were kept in darkness and high humidity for 24 h, and then allowed to grow normally for 3 to 7 days. The incidence of disease at the inoculation site was observed daily. When leaf spots became apparent, spot photography and area measurements were conducted. For *F. graminearum*, conidium suspensions were prepared by culturing the fungus in a CMC liquid medium at 25 °C and shaking at 200 r/min. The concentration of conidia was ensured to be more than 10^6^/mL. Wild-type B73 and mutant maize plants at the 7-leaf stage were punctured at the stem base, and 200 µL of the conidial suspension was inoculated onto each stem segment. After 3 days, the inoculated stems were dissected to observe disease symptoms and calculate the area of disease spots.

## 3. Results

### 3.1. Identification of Maize ACX Family Gene Members

Through bioinformatics predictive analysis, six members of the ZmACX family were identified in the maize genome: *Zm00001d045251*, *Zm00001d052931*, *Zm00001d048890*, *Zm00001d045606*, *Zm00001d042884*, and *Zm00001d003744*. These were named *ZmACX1*, *ZmACX2*, *ZmACX3*, *ZmACX4*, *ZmACX5*, and *ZmACX6*, respectively, in reverse order based on their gene IDs. An analysis of their physical and chemical properties and chromosomal localization revealed that the number of amino acids encoded by the maize ACX family genes ranged from 583 to 696, with molecular weights ranging from 64.78 to 77.66 kDa. *ZmACX4* had the longest amino acid sequence and largest molecular weight, while ZmACX6 had the shortest and smallest molecular weight. The theoretical isoelectric points of the proteins encoded by the maize ACX family genes ranged from 7.26 to 9.29 (Table 1). The maize ACX gene family members were located on four chromosomes: *ZmACX1* and *ZmACX4* on chromosome 9, *ZmACX2* and *ZmACX3* on chromosome 4, *ZmACX6* on chromosome 2, and *ZmACX5* on chromosome 3 (Figure S1).

### 3.2. Evolutionary Analysis of the Maize ACX Family Gene System

**Figure 1:**
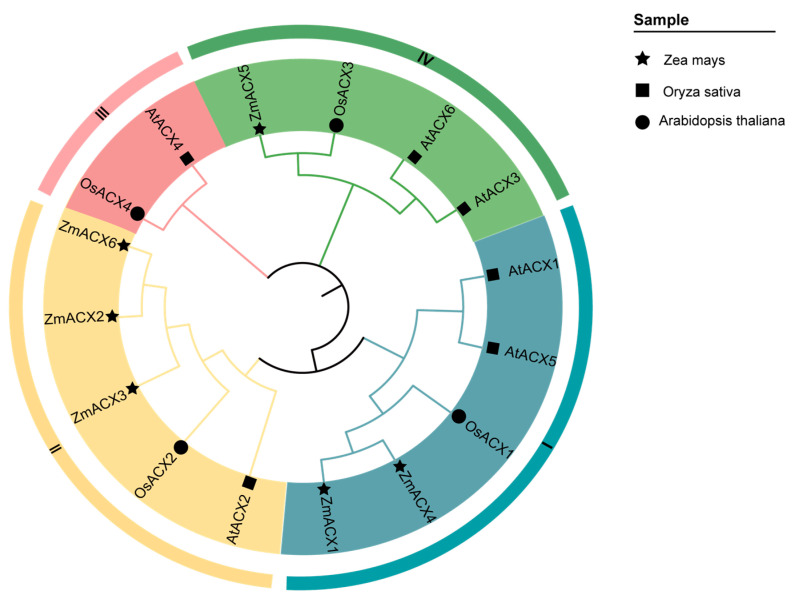
Phylogenetic relationships of the ACX gene family in maize, rice, and Arabidopsis. The nodes represent bootstrap values from 1000 replicates. The four major clades (I–IV) are distinguished by different branches and outer circle colors.

A phylogenetic analysis of the amino acid sequences of ACX family genes from maize, Arabidopsis, and rice revealed that the ACX family genes of Arabidopsis and rice can be divided into four subfamilies: Clade-I, Clade-II, Clade-III, and Clade-IV. The maize ACX family members were distributed among Clade-I, Clade-II, and Clade-IV subfamilies, with Clade-II being the most populated and Clade-IV the least (Figure 1).

### 3.3. Conserved Domain and Conserved Motif Analysis of Maize ACX Family Genes

**Figure 2:**
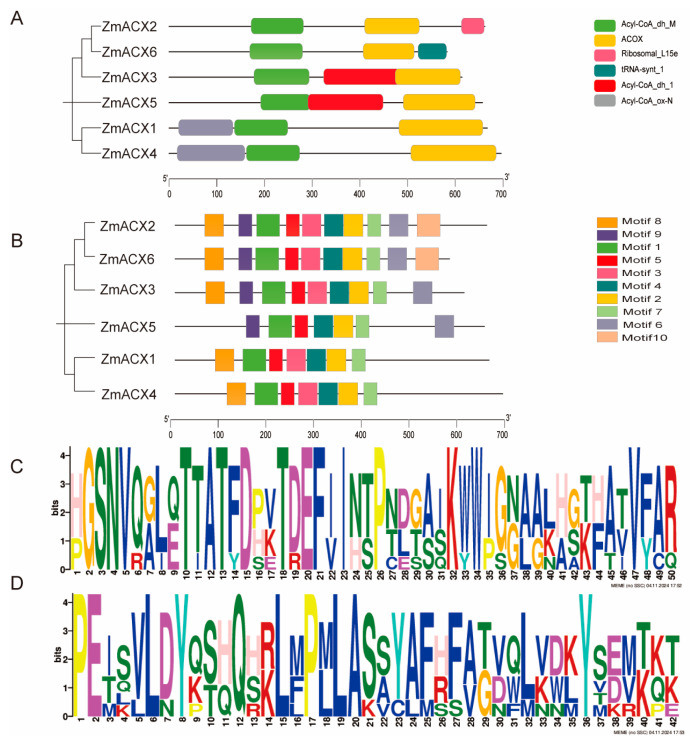
Conserved domains and conserved motifs of ZmACX family members. (**A**) The phylogenetic tree of the ZmACX family is shown. Different color patterns represent different conserved structural domains. (**B**) Conservative motif analysis was performed on ZmACX family members, and the results were visualized. (**C**) Motif 1 sequence. (**D**) Motif 2 sequence.

A predictive analysis of conserved domains was performed on the six identified maize ACX family members using the SMART online website. The results showed that all six members contained ACOX domains and Acyl-CoA_dh_M domains (Figure 2A), indicating that the maize ACX family is highly conserved in evolution.

A conserved motif analysis was performed on the maize ACX family members using MEME online, and the motifs were visualized using TBtools (Figure 2B). The results showed that the maize ACX protein sequence contained 10 conserved motifs, named Motif 1 to Motif 10. Motif 1, Motif 2, Motif 4, Motif 5, and Motif 7 were present in all members of the maize ACX family, suggesting that these motifs are conserved within the family. Among these conserved motifs, Motif 1 and Motif 2 were the most conserved (Figure 2C,D). Additionally, Motif 3 and Motif 8 were found in all members except ZmACX5; all other members except ZmACX1 and ZmACX4 contained Motif 6 and Motif 9; however, only ZmACX2 and ZmACX6 contained Motif 10. Overall, despite some differences, the ZmACX gene family exhibited a high degree of conservation.

### 3.4. Cis-Acting Elements Analysis of Maize ACX Family

**Figure 3:**
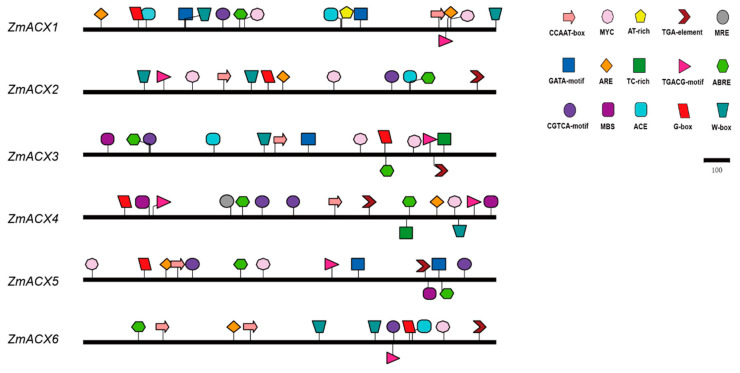
Promoter region cis-acting elements of ZmACX family members. Different colors and shapes represent different cis-acting elements.

In this experiment, the sequence of the maize ACX promoter (upstream 2000 bp) was analyzed, and the composition of the cis-acting elements was predicted using the Plant CARE online tool and then visualized with IBS software (Figure 3). The results showed that the promoter region of the maize ACX gene contained heat stress elements (CCAAT-box, AT-rich element), plant hormone response elements (TGACG-motif, CGTCA-motif, TGA-element, and ABRE), drought response elements (MBS, MYC), light response elements (G-box, ACE, and GATA-motif), and defense response-related cis-acting elements (TC-rich repeats, W-box, MRE, ARE). The promoter region of all maize ACX family members contained the following components: the AT-rich element, plant hormone response elements TGACG-motif, CGTCA-motif, and ABRE, the drought response element MYC, and the light response element G-box. The defense and stress response regulatory elements TC-rich repeats, W-box, MRE, and ARE were also present in most members, suggesting that the maize ACX gene family plays a role in stress response.

### 3.5. Tissue Expression Specificity Analysis of Maize ACX Family Genes

**Figure 4:**
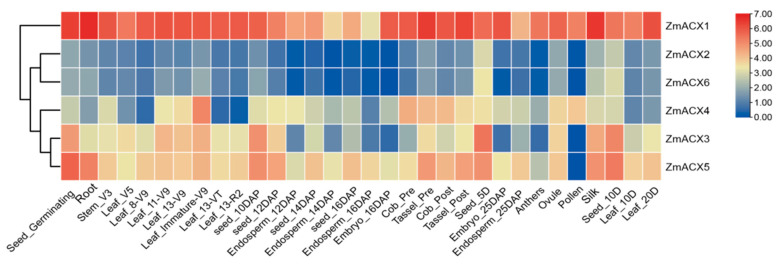
Expression levels of ZmACX family members in different tissues. The gene expression values are presented on a logarithmic scale with a base of 2. The color marks indicate changes in gene expression. Red represents high expression and blue represents low expression.

RNA-Seq data from different maize tissues in the NCBI SRA database were analyzed and visualized using TBtools (Figure 4). The results showed that the expression levels of different genes varied significantly across different growth and development stages and tissues, indicating tissue specificity. *ZmACX1* was highly expressed in all tissues, particularly in roots, stems, leaves, seeds, embryos, tassels, silks, and pollen. This suggests that *ZmACX1* plays a crucial role throughout maize growth and development. Additionally, *ZmACX5* was highly expressed in roots, stems, leaves, and ears, while *ZmACX3* was prominently expressed throughout the seed germination process. *ZmACX4*, on the other hand, demonstrated relatively high expression during another specific stage, suggesting that these genes may play a crucial role in the reproduction and growth of maize. Conversely, the expression levels of *ZmACX2* and *ZmACX6* were consistently low across various stages and tissues, and their expression patterns were strikingly similar.

### 3.6. Analysis of Expression Patterns of Maize ACX Family Genes Under Different Abiotic and Biotic Stresses

**Figure 5:**
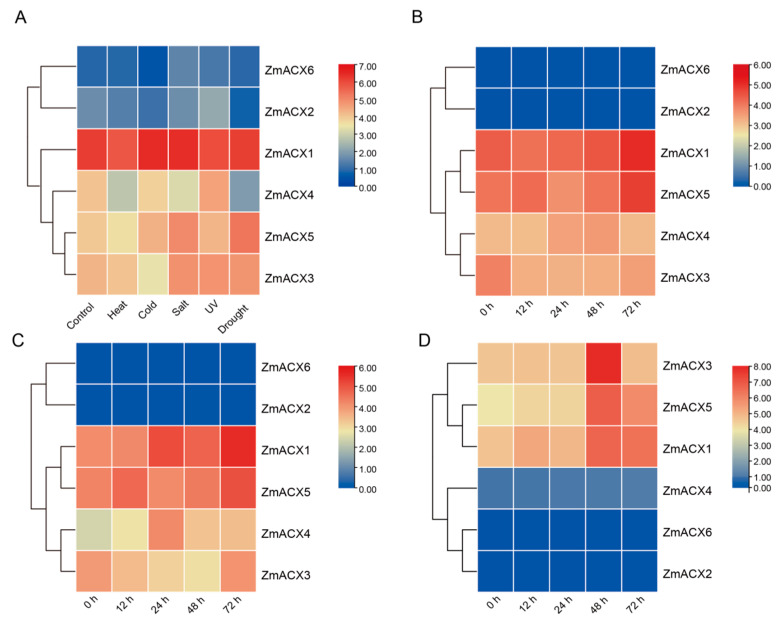
Expression levels of ZmACX family members under abiotic and biotic stresses. (**A**) Abiotic stress heat map of ZmACX family genes; (**B**) expression heat map of ZmACX family genes following *F. verticillioides* infection; (**C**) expression heat map of ZmACX family genes following *A. flavus* infection; (**D**) expression heat map of ZmACX family genes following *F. graminearum* infection. The logarithm of the gene expression value is based on 2. Red represents high expression, and blue represents low expression.

The expression analysis of maize ACX family genes under various abiotic stresses (high temperature, low temperature, salt, UV, and drought) (Figure 5A) revealed that all members, except *ZmACX2* and *ZmACX6*, exhibited decreased expression under high-temperature stress. Under low-temperature stress, only *ZmACX1* showed increased expression, while the others either decreased or remained unchanged. In response to salt stress, only *ZmACX4* demonstrated decreased expression, whereas the others increased or showed no significant change. Under UV stress, *ZmACX1* alone showed a significant decrease in expression. During drought stress, *ZmACX5* was notably affected. It is worth mentioning that *ZmACX1* maintained high expression across all five stress conditions, while *ZmACX2* and *ZmACX6* showed minimal and almost undetectable expression under these stresses. The expression levels of *ZmACX1*, *ZmACX3*, *ZmACX4*, and *ZmACX5* varied to different extents under different abiotic stresses, suggesting their potential roles in responding to high temperature, low temperature, salt, UV, and drought stress.

To investigate the role of maize ACX family genes in the response to pathogen infection, we analyzed maize RNA-Seq data following infection with *F*. *verticillioides*, *A*. *flavus*, and *F*. *graminearum*. The results indicated that *ZmACX1* and *ZmACX5* showed continuously high expression during *F*. *verticillioides* infection, reaching peak levels 72 h post-infection (hpi) (Figure 5B). *ZmACX3* expression initially decreased and then increased after 72 hpi. Conversely, *ZmACX4* expression increased initially, decreased subsequently, and returned to baseline levels, exhibiting an inducible expression pattern. *ZmACX2* and *ZmACX6* showed low and relatively unchanged expression during *F*. *verticillioides* infection. These findings suggest that *ZmACX1*, *ZmACX3*, *ZmACX4*, *and ZmACX5* may be involved in the response to *F*. *verticillioides* stress.

The expression level of maize ACX family genes during *A. flavus* infection was similar to that of *F. verticillioides* infection. *ZmACX1* and *ZmACX5* maintained high expression, reaching peak levels of 72 hpi, although *ZmACX1* expression continued to increase, differing from the pattern observed in *F*. *verticillioides* infection (Figure 5C). *ZmACX3* expression decreased initially and then returned to baseline levels 72 hpi. *ZmACX4* exhibited a clear inducible expression trend, gradually increasing from low initial expression to peak levels at 24 hpi. *ZmACX2* and *ZmACX6* showed low and relatively unchanged expression during *A. flavus* infection. These results suggest that *ZmACX1*, *ZmACX3*, *ZmACX4*, and *ZmACX5* may play a role in response to *A. flavus* stress.

Finally, the analysis of maize RNA-Seq data following *F. graminearum* infection revealed that *ZmACX1*, *ZmACX3*, and *ZmACX5* showed a clear up-regulation trend, with peak expression levels reached 48 hpi, suggesting their potential role in resisting *F. graminearum* infection (Figure 5D). *ZmACX2*, *ZmACX4*, and *ZmACX6* showed low and relatively unchanged expression during *F*. *graminearum* infection.

### 3.7. Analysis of Expression Patterns of Maize ACX Family Genes Under MeJA and SA Treatments

**Figure 6:**
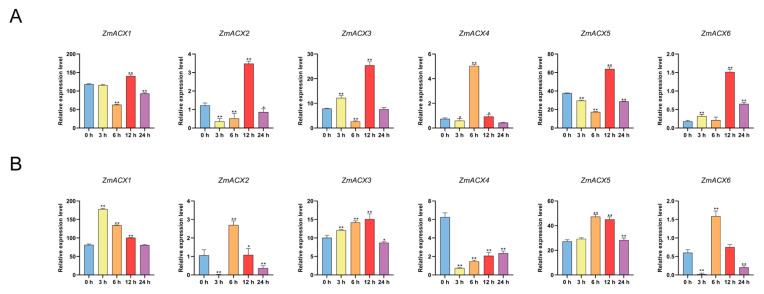
Expression levels of maize ACX family genes treated with exogenous hormones. (**A**) Expression levels of ZmACX family genes following MeJA treatment. Horizontal coordinates represent processing time. Experiments were repeated three times with similar results. Error bars indicate standard deviations. Asterisks indicate significant differences as assessed by Student’s *t*-tests (*, *p* < 0.05; **, *p* < 0.01). (**B**) Expression levels of ZmACX family genes following SA treatment. Horizontal coordinates represent processing time. Experiments were repeated three times with similar results. Error bars indicate standard deviations. Asterisks indicate significant differences as assessed by Student’s *t*-tests (*, *p* < 0.05; **, *p* < 0.01).

Real-time fluorescence quantitative PCR was used to assess ACX family gene expression during maize treatments with MeJA and SA. The results showed that *ZmACX3*, *ZmACX4*, and *ZmACX6* exhibited an induction and up-regulation trend. *ZmACX3* and *ZmACX6* reached peak expression levels 12 h after MeJA treatment, while *ZmACX4* peaked 6 h after hormone treatment. Meanwhile, *ZmACX1*, *ZmACX2*, and *ZmACX5* were down-regulated initially but significantly up-regulated after 12 h of MeJA treatment (Figure 6A). The results of SA treatment indicated that maize ACX family gene expression under SA treatment was opposite to that under MeJA treatment. For example, *ZmACX1*, *ZmACX2*, and *ZmACX6* were down-regulated at 6 h and up-regulated at 12 h under MeJA treatment but were up-regulated at 6 h and down-regulated at 12 h under SA treatment. *ZmACX3* and *ZmACX5* were down-regulated at 6 h under MeJA treatment but up-regulated at 6 h under SA treatment. *ZmACX4* was significantly up-regulated 6 h after MeJA treatment but significantly down-regulated 6 h after SA treatment. Comprehensive analysis showed that, except for *ZmACX4*, which exhibited a significant down-regulation trend, the other genes showed induced up-regulation (Figure 6B).

### 3.8. Disease Resistance Analysis of Maize ACX Family Gene Mutants

**Figure 7:**
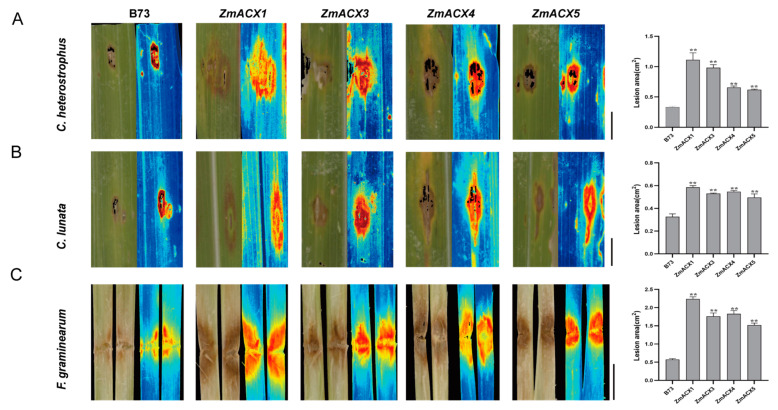
Disease resistance analysis of maize ACX family gene mutants. (**A**) Symptoms and spot area statistics for mutants and wild type inoculated with *C. heterostrophus*. (**B**) Symptoms and spot area statistics for mutants and wild type inoculated with *C. lunata*. (**C**) Symptoms and spot area statistics for mutants and wild type inoculated with *F. graminearum*. Scale bar, 1 cm. *n* = 3. Error bars indicate the mean ± standard deviation. Asterisks indicate significant differences as assessed by Student’s *t*-tests (**, *p* < 0.01).

To elucidate the function of maize ACX family genes in disease resistance, four genes (*ZmACX1*, *ZmACX3*, *ZmACX4*, and *ZmACX5)* were selected for disease resistance analysis of mutants based on their expression in different tissues and under abiotic and biotic stresses. The disease resistance of maize inbred line B73 (wild-type, WT) and *ZmACX1*, *ZmACX3*, *ZmACX4*, and *ZmACX5* mutant plants was assessed following inoculation with *C. heterostrophus*, *C. lunata*, and *F. graminearum*. The results showed that after inoculation with *C. heterostrophus* (Figure 7A) and *C. lunata* (Figure 7B) on the leaves, both wild-type and mutant plants exhibited visible disease spots at the inoculation sites, with mutant symptoms significantly more severe than those of the wild type. Quantitative analysis using Image J software revealed that the spot area of the mutants was significantly larger than that of the wild type. Similarly, the stems of wild-type and mutant plants were inoculated with *F. graminearum* (Figure 7C). The results showed that both wild-type and mutant plants exhibited visible disease spots at the inoculation sites, with mutant symptoms more severe than those of the wild type. Quantitative analysis using Image J software revealed that the lesion area of the mutants was significantly larger than that of the wild type. These findings suggest that *ZmACX1*, *ZmACX3*, *ZmACX4*, and *ZmACX5* play a crucial role in maize resistance to small spots, *Curvula* leaf spot, and stem rot diseases.

## 4. Discussion

In recent years, the ACX family has been identified in numerous species; however, no ACX family members have been reported in maize until now. In this study, bioinformatics technology was employed to identify six *ZmACX* genes in the maize genome: *ZmACX1* (*Zm00001d045251*), *ZmACX2* (*Zm00001d052931*), *ZmACX3* (*Zm00001d048890*), *ZmACX4* (*Zm00001d045606*), *ZmACX5* (*Zm00001d042884*), and *ZmACX6* (*Zm00001d003744*). Conserved domain analysis revealed that all six members contain the conserved ACOX domain, which is crucial for *ACX* gene function. Previously, six and four ACX family genes were identified in Arabidopsis and rice, respectively, and were classified into four subfamilies (I, II, III, and IV) [29]. Our cluster analysis of the six maize ACX members aligned with this previous classification, placing the maize ACX members in subgroups I, II, and IV.

Plant ACX gene expression changes are a major factor in initiating signal sensing and transduction processes in response to environmental stress. H_2_O_2_, the final product of the β-oxidation pathway directly involved in the *ACX* gene, triggers oxidation reactions that activate plant stress response-related signal molecules. For instance, silencing the *GhACX16* gene in upland cotton seedlings conferred high-temperature tolerance, while the overexpression of the *GhACX* gene in thale cress enhanced drought and salt stress tolerance. These findings suggest that ACX plays a vital role in responding to high temperature and salt stress [30]. In this study, we analyzed the cis-acting elements of maize ACX family members and found that they contain various elements related to plant growth, development, and stress response. These include heat stress, drought response, light response, and plant hormone response elements and binding sites of abiotic stress transcription factors such as W-Box (WRKY binding site), ABRE, MYB, MBS, and ARE. This suggests that the maize ACX gene family is crucial for stress response and maize growth and development.

The expression analysis of maize ACX family genes under various abiotic stresses (high temperature, low temperature, salt, UV, and drought) revealed that *ZmACX1* maintained high expression under all five conditions. Additionally, the expression levels of *ZmACX1*, *ZmACX3*, *ZmACX4*, and *ZmACX5* varied under different abiotic stresses, indicating their potential roles in responding to these stresses.

Furthermore, β-oxidation not only degrades fatty acids, but also generates hormone signaling molecules such as IAA, JA, and SA, which activate hormone metabolism in plants, stimulate gene expression, and participate in environmental stress responses [31]. JA serves as a crucial signaling molecule in plants and functions as a “master switch” in both plant development and adaptation to abiotic and biotic stresses. In Arabidopsis, *AtACX1* and *AtACX5* are involved in JA synthesis [21,22], while *OsACX1* in rice may be involved in JA biosynthesis induced by injury [24]. Similarly, tomato *SlACX1* and tea tree *CsACX1* and *CsACX3* are related to JA biosynthesis and respond to injury and disease stress [23,26]. In this study, the expression levels of maize ACX family genes after MeJA treatment were analyzed by qRT-PCR technology. The results showed that the expression levels of maize ACX family genes were significantly up-regulated after MeJA treatment, indicating that maize ACX family genes are involved in JA-mediated plant defense responses. However, the functions of maize ACX family genes in the JA signaling pathway still require further research for confirmation.

ACX is involved in many important pathways in plants. The enhanced activities of AtACX2 and AtACX3 in *Arabidopsis* can improve the plant’s defense ability against necrotizing pathogen infections [13]. After tea plants are invaded by Cgl, the expression of CsACX3 will increase rapidly, thereby increasing the activity of the ACX enzyme in tea plants [26]; OsACX1 in rice can also enhance the defense ability of rice against rice blast [24]. Similarly, our analysis of the disease resistance of the ZmACX1, ZmACX3, ZmACX4, and ZmACX5 mutants revealed that after inoculation with *C. heterostrophus*, *C. lunata*, and *F. graminearum*, the susceptibility of the mutants was significantly more severe than that of the wild type. This indicates that these genes may be involved in the resistance mechanism of maize to pathogenic bacteria. These findings not only provide a new perspective for the functional study of the ACX family genes in maize under biological stress, but also lay an important foundation for the further exploration of the resistance mechanism in maize.

To sum up, the ACX gene plays an important role in the molecular regulatory network of plant stress resistance. This study speculates that maize ACX enables plants to activate effective defense responses against pathogens through the JA pathway network, and among different plant hormones, the relationship between defense responses and growth and development is balanced through synergistic or antagonistic effects. The specific mechanism of action still requires further research.

## 5. Conclusions

The maize ACX family comprises six members featuring highly conserved ACOX domains. The expression patterns of these ACX family members vary significantly across different tissues in response to abiotic and biological stress, as well as under MeJA and SA treatment. Furthermore, mutants of the *ZmACX1*, *ZmACX3*, *ZmACX4*, and *ZmACX5* genes exhibited heightened susceptibility to the pathogenic fungi *C. heterostrophus*, *C. lunata*, and *F. graminearum*. These findings indicate that *ZmACX1*, *ZmACX3*, *ZmACX4*, and *ZmACX5* play a crucial positive regulatory role in maize’s resistance to these pathogenic fungi.

## Figures and Tables

**Table 1:** Physicochemical properties of members of the ZmACX family.

Gene Name	Gene ID	Chr.	Start	End	CDS (bp)	AA	MV (Da)	pI
*ZmACX1*	*Zm00001d045251*	9	17052618	17061922	6034	667	74206.07	7.26
*ZmACX2*	*Zm00001d052931*	4	205248288	205258385	7070	662	73521.80	9.29
*ZmACX3*	*Zm00001d048890*	4	207028046	207034703	5016	614	67543 40	8.52
*ZmACX4*	*Zm00001d045606*	9	28546971	28571638	21667	696	77657.01	7.35
*ZmACX5*	*Zm00001d042884*	3	182845257	182853433	5176	657	73040.00	7.92
*ZmACX6*	*Zm00001d003744*	2	57098888	57107301	5413	583	64778.34	9.24

## Data Availability

Relevant data and materials from this study have been incorporated into the article or provided as Supplementary Information. Please contact the corresponding author if you have any additional questions about this study.

## Supplementary Materials

The following supporting information can be downloaded at: https://www.mdpi.com/article/10.3390/genes16050486/s1, Table S1: qRT-PCR primer sequence; Table S2: DNA identification primer sequence; Figure S1: Chromosome localization of members of the ZmACX family; Molecular identification of mutant plants of *Zmacx1*, *Zmacx3*, *Zmacx4*, and *Zmacx5*. Figure S2: Molecular identification of mutant plants of *Zmacx1*, *Zmacx3*, *Zmacx4*, and *Zmacx5*. (A) DNA level identification; M: Marker (2000bp), 1: *Zmacx1*-F+ *Zmacx1*-R; 2: *Zmacx1*-F+ MU; 3: MU+ *Zmacx1*-R; 4: *zmacx3*-F+ *Zmacx3*-R,; 5: *Zmacx3*-F+ MU; 6: MU+ *Zmacx3*-R; 7: *Zmacx4*-F+ *Zmacx4*-R; 8: *Zmacx4*-F+ MU; 9: MU+ *Zmacx4*-R; 10: *Zmacx5*-F+ *Zmacx5*-R; 11: *Zmacx5*-F+ MU; 12: MU + *Zmacx5*-R; (B) RNA level identification; Expression level validation of *Zmacx1*, *Zmacx3*, *Zmacx4* and *Zmacx5* mutants, using gene-specific primers for qRT-PCR assay, Expression level validation of *Zmacx1*, *Zmacx3*, *Zmacx4*, and *Zmacx5* mutants, using gene-specific primers for qRT-PCR assay, Data are expressed, as mean ± SE of three independent experiments. * and **, significant at *p* < 0.05 and *p* < 0.01, Data are expressed, as mean± se of three independent experiments. * and **, significant at *p* < 0.05 and *p* < 0.01, respectively by the student’s *t*-test.
