# Supplementary material for: Molecular Characterization of Acyl-CoA Oxidase (ACX) Family Genes in Maize Reveals Their Role in Disease Resistance
Source: Genes (Basel). 2025 Apr 25;16(5):486. doi: 10.3390/genes16050486 (PMC12110984; doi:10.3390/genes16050486)
Supplement: Supplementary file 1 [file genes-16-00486-s001.zip › genes-3578140-supplementary.pdf]

Supplementary Materials:

Table S1 qRT-PCR primer sequence

Gene name	Primer name	Sequence (5 '→3')
Tubulin	Q-*Tubulin*-F<br>Q-*Tubulin*-R	CTACCTCACGGCATCTGCTATGT<br>GTCACACACACTCGACTTCATCC
ZmACX1	Q-*ZmACX1*-F	TCATTGTGCAACTGCGAAGC
	Q-*ZmACX1*-R	CTGCGACTATTGTCTGGCGA
ZmACX2	Q-*ZmACX2*-F	CACCAGCACAAACTGATGCC
	Q-*ZmACX2*-R	TGTGTGATTCTGCGAGCGTA
ZmACX3	Q-*ZmACX3*-F	ACTCACGCTGTTCTTCCTGG
	Q-*ZmACX3*-R	GCAGCAGAGCATACCTCACA
ZmACX4	Q-*ZmACX4*-F	TACAGCGCAGTCAGAAAGCA
	Q-*ZmACX4*-R	CCATGTCCACCACAGAGCTT
ZmACX5	Q-*ZmACX5*-F	TCCGCTTACACTTGGTCGAG
	Q-*ZmACX5*-R	GTGTCGCCTTCAGAGCACTA
ZmACX6	Q-*ZmACX6*-F	CACCAGCACAAACTGATGCC
	Q-*ZmACX6*-R	TATTCCACGCACCGAAACCT

Table S2 DNA identification primer sequence

Gene name	Primer name	Sequence (5 '→3')
Mu67	*Mu67*	GAAGCCAACGCCAWCGCCTCY<br>ATTTCGTCGAAT
ZmACX1	*zmacx1*-F	GGATGTTTTCACGAAACCTGACCA
	zmacx1-R	TACAGGAGATCAGATGCGGAAAG
ZmACX3	*zmacx3*-F	CATGTCATCAGGGTCATAC
	zmacx3-R	TACTGGACGCCGAGCTTGAC
ZmACX4	*zmacx4*-F	CTGCAGGTTTCTATGAGCGGT
	zmacx4-R	CAATGCATACAGGCTAAGCCCA
ZmACX5	*zmacx5*-F	AGCAATCCCGTCGATCACCCAG
	zmacx5-R	GTGGTACACACTAATGGCAGGTCC

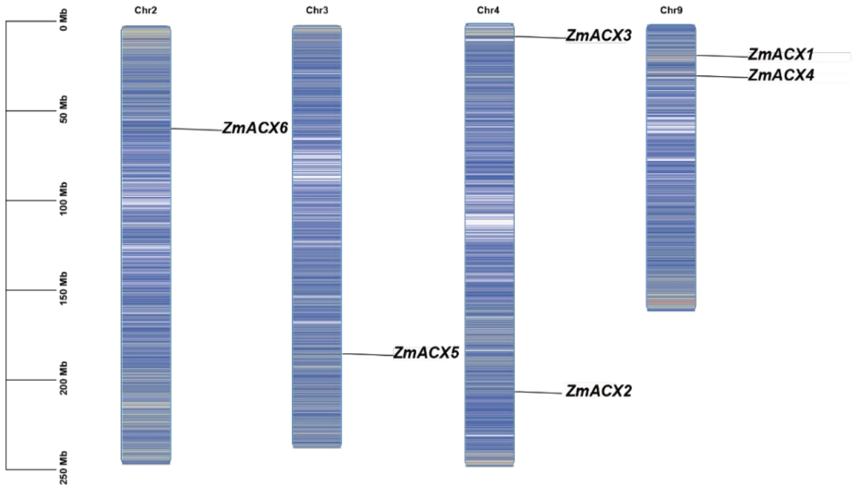

Figure S1. Chromosome localization of members of the ZmACX family. The blue lines on the chromosome represent other genes in the maize genome. A higher number of blue lines indicates greater gene density, while fewer blue lines indicate lower gene density.

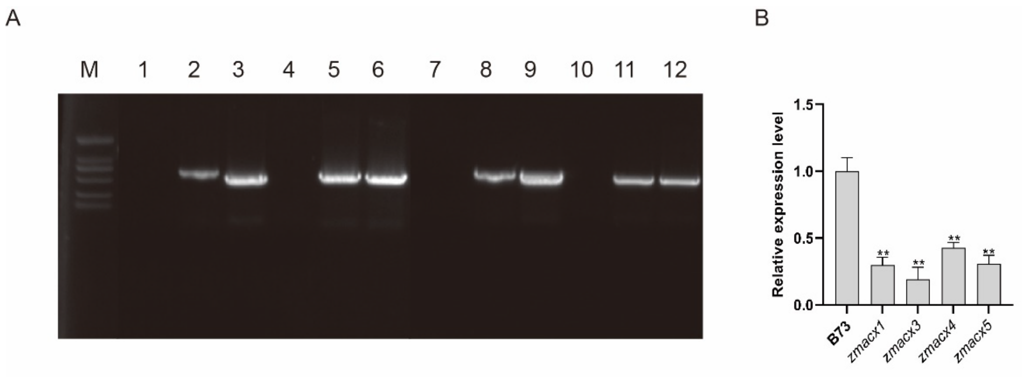

Figure S2. Molecular identification of mutant plants of *zmacx1*, *zmacx3*, *zmacx4*, and *zmacx5*. (A) DNA level identification; M: Marker(2000bp), 1: *zmacx1*-F+ *zmacx1*-R; 2: *zmacx1*-F+ MU; 3: MU+ *zmacx1*-R; 4: *zmacx3*-F+ *zmacx3*-R,; 5: *zmacx3*-F+ MU; 6: MU+ *zmacx3*-R; 7: *zmacx4*-F+ *zmacx4*-R; 8: *zmacx4*-F+ MU; 9: MU+ *zmacx4*-R; 10: *zmacx5*-F+ *zmacx5*-R; 11: *zmacx5*-F+ MU; 12: MU + *zmacx5*-R; (B) RNA level identification; Expression level validation of *zmacx1*, *zmacx3*, *zmacx4* and *zmacx5* mutants, using gene-specific primers for qRT-PCR assay, Expression level validation of *zmacx1*, *zmacx3*, *zmacx4*, and *zmacx5* mutants, using gene-specific primers for qRT-PCR assay, Data are expressed, as mean±SE of three independent experiments. * and **, significant at P<0.05 and P<0.01, Data are expressed, as mean± se of three independent experiments. * and **, significant at P<0.05 and P<0.01, respectively by the student's t-test.
